# Effect of Catechins on Upper Respiratory Tract Infections in Winter: A Randomized, Placebo-Controlled, Double-Blinded Trial

**DOI:** 10.3390/nu14091856

**Published:** 2022-04-29

**Authors:** Naoki Ozato, Tohru Yamaguchi, Tatsuya Kusaura, Hidefumi Kitazawa, Masanobu Hibi, Noriko Osaki, Takahiro Ono

**Affiliations:** 1 Health & Wellness Products Research Laboratories, Kao Corporation, Tokyo 131-8501, Japan; yamaguchi.tohru@kao.com (T.Y.); kusaura.tatsuya@kao.com (T.K.); osaki.noriko@kao.com (N.O.); 2 Biological Science Research Laboratories, Kao Corporation, Tokyo 131-8501, Japan; kitazawa.hidefumi@kao.com (H.K.); hibi.masanobu@kao.com (M.H.); 3 Ueno-Asagao Clinic, Tokyo 110-0015, Japan; t.ono@ueno-asagao.clinic

**Keywords:** catechin, clinical trial, influenza-like illness, physical symptoms, randomized, placebo-controlled, double-blinded trial

## Abstract

Tea catechins are plant-derived compounds that improve immune functions. Previous randomized control trials have demonstrated the efficacy of primarily epi-type catechins against upper respiratory tract infections (URTIs). Green tea can be consumed in several ways, including popular bottled beverages. These beverages, however, require sterilization during manufacturing, which results in catechin isomerization. We conducted a randomized, double-blinded, placebo-controlled trial involving healthy Japanese participants to evaluate whether catechin consumption via bottled beverages has an alleviating effect on the duration and severity of URTIs in winter. The catechin group (490 mg catechin, 0.14%, containing 59% epi-type catechin, *n* = 55) showed reduced durations of running nose, nasal congestion, and headache, compared with the placebo group (0 mg catechin, *n* = 54; *p* = 0.013, 0.018, and <0.001, respectively). Furthermore, when considering physical symptoms, the duration of nasopharyngeal symptoms improved significantly in the catechin group (*p* < 0.001) compared with that in the control group. The daily consumption of catechin thus reduced the duration and severity of URTIs in healthy men and women. Humans are regularly exposed to several potential infectious threats, and the oral administration of heat-epimerized tea catechins might help prevent and reduce the severity of URTIs.

## 1. Introduction

The recent influenza [1,2] and SARS-CoV-2 [3] pandemics have significantly increased global interest in preventive measures against infectious diseases. Given the unpredictable nature of these epidemics and/or pandemics, devising strategies to reduce their impact is of utmost importance [2]. Acute upper respiratory tract infections (URTIs) are the most common infectious diseases caused by various viruses. Non-pharmaceutical interventions (NPIs) aimed at mitigating the transmission of viral respiratory infections include frequent washing of hands, wearing masks, physical distancing, and gargling, which are commonly followed globally. Randomized control trials (RCTs) have demonstrated the efficacy of handwashing [4] and wearing masks. Consequently, the development of new approaches based on NPIs that are easily accessible to the general population is an important challenge in public healthcare.

Green tea is acknowledged as a healthy drink worldwide, and epidemiological studies have conclusively demonstrated that the consumption of 1–5 cups per day might have a prophylactic effect against influenza [5]. Tea catechins improve immune function, the efficacy of which has been demonstrated in certain RCTs [6,7]. The consumption of catechin and L-theanine capsules for a period of 12 weeks was found to reduce the incidence of cold and flu symptoms and to enhance the interferon-γ responses of peripheral blood mononuclear cells to γδ T-cell antigen stimulation in an American study [6]. A similar effect consequent to the intake of catechins and xanthan gum has also been demonstrated in the Japanese population [7]. Additionally, the protective effect of catechin consumption against viral respiratory infections has been shown by a meta-analysis [8]. These RCTs, thus, collectively indicate that tea catechins contribute to immunity.

Green tea can be consumed in several ways. Of these, bottled or canned beverages are extremely popular forms that have a sizeable market share in Asia. The manufacturing process of these products mandates proper sterilization. The epimerization of tea catechins is well documented, and most previous studies have focused on the beneficial effects of powdered tea that mainly contains the epi-type of catechins [6,7]. The proportion of non-epi to epi-type catechins is halved during the manufacturing of bottled green tea [9,10], and heat-epimerized tea catechins have been reported to exert an effect distinct from that of the original catechins in some studies [11,12]. Therefore, whether heat-epimerized tea catechins have similar beneficial effects as powdered tea is unknown to date.

Here, we conducted a randomized, double-blinded, placebo-controlled trial involving healthy Japanese participants to evaluate the preventive effect of the consumption of bottled beverages containing catechin against the onset and progression of viral respiratory infections in winter.

## 2. Materials and Methods

### 2.1. Participants

In total, 109 volunteers (55 men and 54 women; mean age ± SD: 44.5 ± 13.5 years) participated in this trial. The inclusion criterion included healthy individuals between 20–65 years of age who were not undergoing treatment for allergic rhinitis during the study period. The exclusion criteria were as follows: ongoing treatment for severe disease; history or indications of cardiac or cerebrovascular disease; history of coronavirus infection; use of therapeutic medication for diabetes, dyslipidemia, or hypertension; history of heavy drinking; anemia; food allergies; and hypersensitivity to the test beverages. Furthermore, we excluded those with propensity for seasonal allergy symptoms during the test period and were receiving medication. The influenza vaccine was a factor that greatly influenced the results of this study. In this study, we recruited volunteers who had not been vaccinated against influenza. Regarding supplements, participants taking catechin as a supplement were excluded. However other supplements currently in use may be continued during the test period. On the other hand, it was prohibited to take new supplements. Furthermore, during the study period, vaccination against coronavirus had not yet commenced in Japan. Informed written consent was obtained from all participants included in the study. The target sample size was set at 100 subjects by referring to a previously conducted trial that determined the efficacy of capsules containing epigallocatechin gallate and theanine, the main components of catechins [6], although the dosage is not known.

### 2.2. Test Beverages

Catechins were extracted from green tea via the hot water extraction method [10]. Caffeine has been previously reported to modulate the secretion of various adipocytokines, including TNF-α and IL-6, in animal studies [13]. This necessitated normalization of the quantity of caffeine in the placebo and catechin beverages. The test beverages (base water) were prepared using a ready-made beverage from Kao Corporation. It was referred to as the “catechin beverage” and contained 490 mg catechins and 10 mg caffeine per 350 mL. The caffeinated placebo beverage contained 0 mg catechins and 10 mg caffeine per 350 mL (Table 1). The catechin beverage contained several catechins, including epigallocatechin gallate (EGCg, 112 mg), epicatechin gallate (ECg, 36 mg), epigallocatechin (EGC, 107 mg), epicatechin (EC, 33 mg), gallocatechin gallate (GCg, 71 mg), catechin gallate (Cg, 15 mg), gallocatechin (GC, 94 mg), and catechin (C, 22 mg). The epi-type catechins, namely EGCg, ECg, EGC, and EC, comprised 59% (287 mg), and the non-epi-type catechins, which were GCg, Cg, GC, and C, constituted 41% (203 mg) of the total catechins. The amount of catechins in the test beverages was measured using HPLC carried out on an L-column ODS (4.6 mm diameter × 250 mm length; Chemicals Evaluation and Research Institute, Tokyo, Japan) [10].

### 2.3. Study Design

The present study was a randomized, double-blinded, placebo-controlled, parallel-group trial that was conducted over a period of 12 weeks. The participants were randomly allocated to the catechin or placebo groups during the screening process. A randomization with stratified permutated block design was performed considering factors such as age, gender, and history of influenza infection. All participants were administered either the catechin or placebo beverage once daily during the experimental period. The participants were additionally interviewed by a doctor and instructed to maintain their usual lifestyle and dietary habits during the study period without any dietary restrictions. As a limitation, participants were instructed to drink no more than one cup (200 mL) of green tea per day during the study, as it might have affected the results of this study. Dietary and medical history of the participants was obtained using a questionnaire.

This study was performed at the Ueno Asagao Clinic (Tokyo, Japan) from 16 February 2021 to 20 May 2021 in accordance with the Declaration of Helsinki and was managed by TES Co., Ltd. (Tokyo, Japan), a contract research organization. All protocols were approved on 4 February 2021, by the Ueno Asagao Clinic Ethics Committee and were registered with the UMIN Clinical Trial Registry before the enrolment of participants (UMIN000043122). The trial was performed in accordance with the Declaration of Helsinki and the trial plan was reviewed and approved by the local ethics committee Ueno Asagao Clinic (Tokyo, Japan, g2020004). Written informed consent was obtained from all participants before their inclusion in the study.

### 2.4. Study Outcome

The primary outcome measures used to analyze the efficacy of the catechin beverage were the duration of URTIs in days and severity of symptoms.

### 2.5. Measurements of Duration of URTIs and Severity of Symptoms

Participants recorded the severity of URTIs, such as cough and fever, and that of common cold symptoms, such as sore throat, runny nose, nasal congestion, sneezing, and headache. They were asked to rate the severity on a scale with five grades—normal, slight, mild, moderate, and severe—daily during the test period via a mobile phone app. For the mobile phone app, the download URL was distributed to participants, and participants were asked to install the app on their mobile phones. At the time of visit, a login ID was assigned individually and linked to the test ID. The incidence of URTIs was determined based on the severity of symptoms, evaluated according to a previously published list of diagnostic criteria [14]. The severity of each symptom was assessed according to the guidelines published in a previous report [15]. Furthermore, they were asked about medication taken during the study period via a mobile phone app.

### 2.6. Physical Symptom Assessment

The mental and physical condition of affected individuals is known to influence the URTIs and its severity [16]; thus, their assessment in the participants was necessary. The General Health Questionnaire (GHQ60), which utilizes a psycho-social approach, was employed to assess the baseline mental condition [17]. The questionnaire analyzes participants on the basis of the following four subscales: (i) somatic symptoms, (ii) anxiety/insomnia, (iii) social dysfunction, and (iv) severe depression.

### 2.7. Statistical Analysis

The characteristics of the study participants are reported as the mean ± standard deviation (SD). At baseline, intragroup comparisons were conducted by performing Fisher’s exact test or a Wilcoxon rank-sum test. Data on the cumulative days of URTIs or the severity of symptoms were presented as incident numbers (*n*). Cumulative durations of each symptom were compared between the catechin and placebo groups using the Fisher’s exact test. Statistical tests were two-sided, and values of *p* < 0.05 were considered statistically significant. All analyses were performed using R software version 4.1.10.

## 3. Results

### 3.1. Participants and Baseline Characteristics

**Figure 1:**
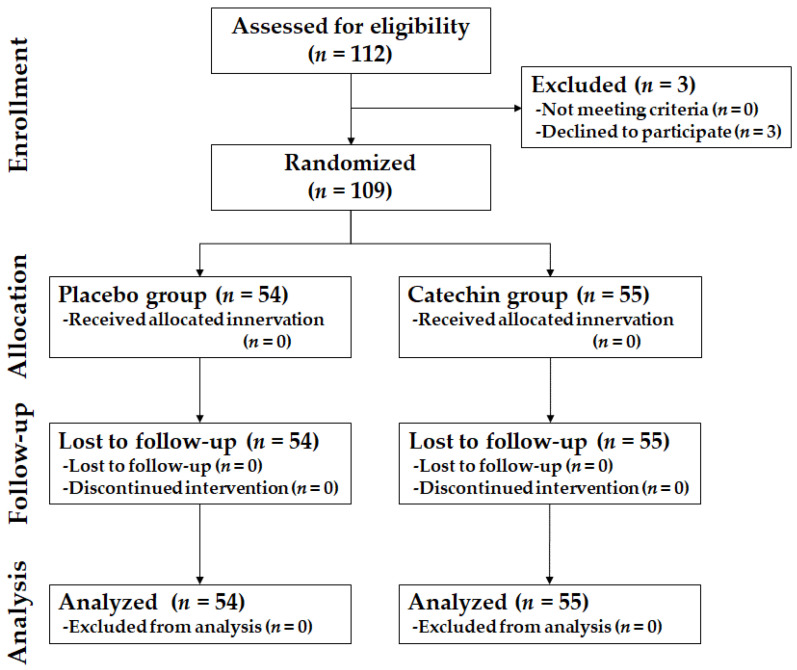
Consolidated standards of trial diagram: enrolment, random assignment, and follow-up of participants.

The recruitment of participants was conducted in two phases. In total, 112 participants were randomized to create two groups of 56 participants each. The study, however, commenced with 109 participants (55 in the catechin and 54 in the placebo group) on account of the withdrawal of three individuals. A flowchart depicting the enrolment, allocation, follow-up, and analyses of participants is shown in Figure 1. All participants completed the trial by complying with the study protocol, which required the consumption of more than 80% of the prescribed test beverage. During the test period, participants were urged to contact the consultation desk when they were in poor physical condition, such as allergic rhinitis; however, there were no such participants. No adverse events were noted in this study. During the study period, six participants in the catechin group and seven participants in the placebo group took some medication. However, no significant differences among these individuals were observed as per Fisher’s exact test.

The baseline characteristics of the participants are shown in Table 2. The mean age of the catechin group (male: 50.9%, female: 49.1%) was 44.8 years and that of the placebo group (male: 50%, female: 50%) was 44.2 years (*p* = 0.844). Of the total participants, 5.5% in the catechin group and 9.3% in the placebo group (*p* = 0.693) reported that they were “currently smoking”. Another 45.5% in the catechin group and 55.6% in the placebo group (*p* = 0.388) confirmed that they regularly engaged in exercise. Additionally, 47.3% in the placebo group and 57.4% in the placebo group (*p* = 0.386) revealed that they consumed green tea “twice per week or more”. A previous history of influenza infection over the past 3–4 years was reported for 21.8% of the catechin group and 16.7% of the placebo group (*p* = 0.661). The proportion of individuals living alone was 32.7% in the catechin group and 25.9% in the placebo group (*p* value = 0.569). Furthermore, the proportion of participants younger than 15 years of age was 32.7% in the catechin group and 25.9% in the placebo group (*p* = 0.569). None of the baseline parameters differed significantly between the placebo (*n* = 54) and catechin groups (*n* = 55). The baseline mental and physical conditions of the participants are presented in Table 3, which shows no significant differences between the two groups in terms of somatic symptoms, anxiety/insomnia, social dysfunction, or severe depression (*p* = 0.805, 0.177, 0.592, and 0.348, respectively). Regarding supplements, six participants were supplement users in the catechin group and five participants were supplement users in the placebo group, and there was no significant difference between the two groups using the Fisher’s exact test.

### 3.2. Effect of Catechins on Duration of URTIs and Severity of Symptoms

The effects of catechins on the incidence and severity of URTIs were evaluated based on the severity of influenza-like symptoms, such as cough and fever, and that of common cold symptoms, such as sore throat, runny nose, nasal congestion, sneezing, and headache. Table 4 shows a comparison of the severity of symptoms using the Fisher’s exact test.

The Catechin group reported 4611 days of “normal” to “mild” and 9 days of “moderate” to “severe” running nose. The placebo group reported 4513 days of “normal” to “mild” and 23 days of “moderate” to “severe” running nose. The catechin group, thus, experienced a significantly lower duration and severity of running nose than those observed in the placebo group (*p* = 0.0127).

Furthermore, the catechin group reported 4616 days of “normal” to “mild” and 4 days of “moderate” to “severe” nasal congestion. The placebo group reported 4522 days of “normal” to “mild” and 14 days of “moderate” to “severe” nasal congestion. The catechin group, thus, also exhibited a significantly lower duration and severity of moderate to severe (*p* = 0.0181) nasal congestion than those observed in the placebo group.

The catechin group reported 4613 days of “none” to “mild” and 7 days of “moderate” to “severe” headache. The placebo group reported 4504 days of “none” to “mild” and 31 days of “moderate” to “severe” headache. Thus, the catechin group also exhibited a significantly lower duration and severity of moderate to severe (*p* < 0.0001) headaches than those observed in the placebo group. No significant differences were observed in the other examined parameters between these groups.

### 3.3. Effect of Catechins on Physical Symptoms

The effect of catechins on the incidence of each physical symptom was evaluated and the discrimination criteria for these are depicted in Table 5. Table 6 compares the severity of each physical symptom based on Fisher’s exact test.

The catechin group reported 9227 days of “none” to “mild” and 13 days of “moderate” to “severe” nasopharyngeal symptoms. The placebo group reported 9035 days of “none” to “mild”, and 37 days of “moderate” to “severe” nasopharyngeal symptoms. Statistical analysis, thus, revealed a significantly reduced duration and severity of moderate and severe nasopharyngeal symptoms in the catechin group compared to those in the placebo group (*p* = 0.0006). No significant differences were observed in the other examined parameters.

## 4. Discussion

In this study, we conducted a randomized, double-blinded, placebo-controlled trial involving healthy Japanese participants. The results showed that catechin consumption could reduce the onset and severity of URTI symptoms in winter. Daily catechin intake reduced the duration and severity of running nose, nasal congestion, and headache in healthy men and women (Table 4). Furthermore, when considering physical symptoms, the duration and severity of nasopharyngeal symptoms improved significantly after administration of the catechin beverage (Table 6). These results suggest that catechin consumption via bottled beverages has a significant preventive effect against the onset and severity of URTI symptoms, which is similar to that conferred by catechin ingested via powders or capsules [6,7]. Green tea can be consumed in numerous ways, and our results could aid in the expansion of catechin consumption via bottled beverages. Bottled beverages contain epi-type and non-epi-type catechins (Table 1), whereas powders and capsules primarily contain epi-type catechins [6,7]. Catechin consumption has been previously shown to significantly enhance NK cell activity in humans at approximately the same isomerization rate and quantity and concentration of catechins as those used in the present study [18]. Furthermore, the powder or capsular form that primarily contains epi-type catechins increases NK cell cytolysis in animal studies [19]. This suggests that while the isomerization rates of catechins differ greatly between bottled and powdered beverages, this might not have a significant effect on their protective effects against URTIs. However, further studies are warranted to establish this unequivocally. Catechins are known to be metabolized by the gut microbiota [20]. Studies have indicated that NK cell activity is not modulated by catechin itself, but rather by metabolites produced by gut microbiota, including 5-(3′,5′-dihydroxyphenyl)-γ-valerolactone (EGC-M5) [21]. EGC-M5 was found to be produced from EGCg and EGC in humans [22]; however, it is unknown whether it is also produced from the isomeric forms of EGCg, EGC, GCg, and GC. Although the metabolism of catechin isomers varies only slightly in vivo [23], it is clear that they affect the efficacy of catechins in terms of immune modulation. Additionally, catechins enhance the activity of secretory IgA apart from activating NK cells [24], as well as having an inactivating effect on viruses, such as influenza [25] and coronavirus [26]. Furthermore, meta-analysis showed that tea gargling has preventive effects against influenza infection and URTIs [8]. However, the concentration of the tea might be important from this point of view, and further investigation is warranted.

The present study was conducted during the ongoing the COVID-19 pandemic [27], during which the participants were presumed to have refrained from venturing outside [27], worn masks, and been mindful about frequently washing their hands [28]. In fact, the duration of symptoms in the control group was lower than that reported in other studies that were conducted before the onset of the COVID-19 pandemic [14,15]. This is significant since it establishes the protective role of catechin consumption without the confounding effects of extraneous factors. Developing new approaches based on NPIs that can be easily used by the general population and made available at workplaces is a significant challenge in public healthcare. Tea catechin consumption might constitute one aspect of a multi-pronged solution to this problem, although further studies are required to unambiguously establish the therapeutic efficacy of these compounds.

This study has certain limitations. Several studies have reported that polyphenols, including catechins, modulate immune function and enhance the robustness of cellular immune responses [19,21,29,30,31,32]; however, we could not determine the effective catechin species or concentration. Furthermore, this study was conducted during the ongoing COVID-19 pandemic, where the behavior of at least a section of the population is expected to have varied drastically between periods of emergency and non-emergency. The effects of catechins on immune function were not elucidated. Finally, we could not assess if the benefits of the catechin beverage were associated with reduced levels of inflammatory markers. Consequently, further long-term, large-scale, double-blinded clinical trials based on the general population are essential to establish the clinical utility of catechins as protective agents against UTRIs.

## 5. Conclusions

In conclusion, we conducted a randomized, double-blinded, placebo-controlled trial and found that the daily intake of heat-epimerized tea catechins could reduce the duration and severity of URTIs in healthy men and women. Further research on the mechanisms underlying the effects of catechins will help to better understand this observation. Given that humans are regularly exposed to several infectious threats, the oral administration of catechins might aid in preventing the onset and alleviating the severity of URTI symptoms in humans.

## Figures and Tables

**Table 1:** Composition of the test beverages.

	Catechin Beverage	Placebo Beverage
Epigallocatechin gallate (mg)	112	0
Epicatechin gallate (mg)	36	0
Epigallocatechin (mg)	107	0
Epicatechin (mg)	33	0
Gallocatechin gallate (mg)	71	0
Catechin gallate (mg)	15	0
Gallocatechin (mg)	94	0
Catechin (mg)	22	0
Total catechins (mg)	490	0
Caffeine (mg)	22	21

**Table 2:** Characteristics of participant groups administered either the placebo (*n* = 54) or catechin (*n* = 55).

Variables		Catechin	Placebo	*p*-Value
Participants	*n*	55	54	
Women proportion *^,a^	%	49.1	50.0	0.9244
Age *^,b^		44.8 ± 13.3	44.2 ± 13.7	0.8438
Smoking rate *^,a^	yes, %	5.5	9.3	0.6934
Exercise habits “yes” *^,a^	yes, %	45.5	55.6	0.3881
Green tea intake “more than twice per week” *^,a^	yes, %	47.3	57.4	0.3857
History of influenza over the past 3–4 years *^,a^	yes, %	21.8	16.7	0.6607
Proportion of those living alone *^,a^	%	32.7	25.9	0.5692
Proportion of those under the age of 15 years *^,a^	%	32.7	25.9	0.5692

*^,a^: Fisher’s exact test was used. *^,b^: Wilcoxon rank-sum test was used and data are presented as mean ± SD.

**Table 3:** Comparison of subscale scores for GHQ60 between the placebo (*n* = 54) and catechin (*n* = 55) groups.

Item	Group	*n*	Score *	*p*-Value ^#^
Somatic symptoms	Catechin	55	1.1 ± 1.4	0.8047
	Placebo	54	1.1 ± 1.6	
Anxiety/insomnia	Catechin	55	1.7 ± 1.9	0.1772
	Placebo	54	1.3 ± 1.7	
Social dysfunction	Catechin	55	0.4 ± 1.2	0.5915
	Placebo	54	0.5 ± 1.2	
Severe depression	Catechin	55	0.3 ± 0.8	0.3475
	Placebo	54	0.1 ± 0.4	
Total score	Catechin	55	8.7 ± 8.7	0.1362
	Placebo	54	7.1 ± 8.9	

* Mean ± SD, ^#^ Wilcoxon test was used.

**Table 4:** Cumulative duration (in days) of grades as scores of severe–moderate and mild–normal for each group.

Symptoms	Group	*n*	Normal, Slight, Mild	Moderate, Severe	*p*-Value	
Chill	Catechin	55	4606	14	0.1047	
	Placebo	54	4512	24		
Feverishness	Catechin	55	4614	6	0.2890	
	Placebo	54	4534	2		
Running nose	Catechin	55	4611	9	0.0127	#
	Placebo	54	4513	23		
Articular pain	Catechin	55	4619	1	0.0678	
	Placebo	54	4530	6		
Nasal congestion	Catechin	55	4616	4	0.0181	#
	Placebo	54	4522	14		
Sore throat	Catechin	55	4614	6	0.1250	
	Placebo	54	4535	1		
Cough	Catechin	55	4619	1	1.0000	
	Placebo	54	4536	0		
Headache	Catechin	55	4613	7	<0.0001	###
	Placebo	54	4504	31		

Numbers represent cumulative duration in days. Fisher’s exact test was used for statistical analysis. Catechin group: *n* = 4620 (55 subjects × 84 days) and placebo group: *n* = 4536 (54 subjects × 84 days). #: *p* < 0.05; ###: *p* < 0.001.

**Table 5:** Discrimination criteria for the analysis of sets of physical symptoms.

Variables	Criteria
Nasopharyngeal symptoms	For the symptoms “running nose” and “nasal congestion”, the total number of “normal”, “slight”, or “mild” days and the total number of “moderate” or “severe” days were tabulated.
Hypo-pharyngeal symptoms	For the symptoms “sore throat” and “cough”, the total number of “normal”, “slight”, or “mild” days, and the total number of “moderate” or “severe” days were tabulated.
Systemic symptoms	For the symptoms “headache”, “chill”, “general malaise”, “feverishness”, and “articular pain”, the total number of “normal”, “slight”, or “mild” days and the total number of “moderate” or “severe” days were tabulated.

**Table 6:** Cumulative duration (in days) for individual sites of symptoms graded as scores of severe–moderate and mild–normal for each group.

Symptoms	Group	*n*	Normal, Slight, Mild	Moderate, Severe	*p*-Value	
Nasopharyngeal symptoms	Catechin	55	9227	13	0.0006	###
	Placebo	54	9035	37		
Hypo-pharyngeal symptoms	Catechin	55	9233	7	0.0703	
	Placebo	54	9071	1		
Systemic symptoms	Catechin	55	23,043	57	0.1158	
	Placebo	54	22,605	74		

Numbers represent the cumulative duration in days. Fisher’s exact test was used for statistical analysis. ###: *p* < 0.001.

## Data Availability

The data supporting the findings of this study can be availed from the corresponding author upon reasonable request.
